# Effectiveness of a Natural-Rubber Anal Dilator (ParaSelf) After Pediatric Anorectal Surgery: A Historical-Controlled Intervention Study

**DOI:** 10.14740/gr2124

**Published:** 2026-04-27

**Authors:** Kwanhathai Sakulsansern, Suppachai Lawanaskol

**Affiliations:** a Department of Surgery, Sawanpracharak Hospital, Nakhon Sawan, Thailand; b Chaiprakarn Hospital, Chiang Mai, Thailand

**Keywords:** Stenosis, Dilation, Anorectal malformations, Hirschsprung disease, Rubber

## Abstract

**Background:**

Anal stenosis is a frequent complication after anorectal reconstruction for anorectal malformation (ARM) or Hirschsprung disease (HD). Conventional rigid dilators are uncomfortable, expensive, and poorly adapted to pediatric anatomy. Natural rubber dilators may offer better conformity and outcomes but have not been evaluated. The aim of the study was to compare the efficacy and safety of ParaSelf, a natural rubber-based anal dilator, with conventional rigid dilators in children after anorectal surgery.

**Methods:**

This retrospective cohort study included 98 children with ARMs or HD. Children who used conventional dilators during 2017–2022 served as the preimplementation control cohort, and children who used ParaSelf during 2022–2025 comprised the post-implementation cohort. The primary endpoint was time to achieve the age-specific target anal diameter, analyzed using multivariable Weibull parametric proportional hazards regression. Secondary endpoints included anal stenosis (including surgery-requiring stenosis), pain, caregiver satisfaction, and major postoperative complications.

**Results:**

Of 98 patients, 33 (33.7%) received ParaSelf. The ParaSelf group reached the target diameter faster than controls (median 5.6 vs. 9.4 months). Weibull regression showed a nearly 10-fold higher hazard of reaching the target diameter with ParaSelf (hazard ratio (HR) = 9.9; 95% confidence interval (CI), 5.2 to 18.7; P < 0.001). Pain scores during home dilation were lower (median difference = −2.58; 95% CI, −3.40 to −1.76; P < 0.001), and caregiver satisfaction was higher (median difference = 2.00; 95% CI, 1.52 to 2.48; P < 0.001) with ParaSelf versus conventional dilators. No irritation, bleeding, leakage, or surgery-requiring stenosis occurred in the ParaSelf group.

**Conclusions:**

ParaSelf was associated with faster dilation, less pain, fewer complications, and higher caregiver satisfaction than conventional dilators and may be a more acceptable alternative for pediatric anorectal reconstruction.

## Introduction

Anorectal malformations (ARM) and Hirschsprung disease (HD) are among the most common congenital colorectal disorders in children and often require definitive anorectal reconstruction early in life [1, 2]. Postoperative anal stenosis remains a frequent and important complication, leading to pain, constipation, difficult defecation, and impaired quality of life, and may necessitate further surgical interventions [2–4]. To prevent stenosis, postoperative anal dilation programs are routinely recommended as a part of standard care [3–5].

Conventional anal dilators are typically rigid, cylindrical devices made from hard plastic, paraffin candle or metal [6, 7]. Although effective in principle, their stiffness, shape, and size increments are often poorly adapted to pediatric anatomy and to the postoperative perineum [6]. Children frequently experience significant pain and discomfort during home dilation, and caregivers may feel anxious to perform the procedure [4, 8, 9]. These problems reduce adherence to the prescribed dilation schedule and limit the effectiveness of conventional dilation programs [4, 6]. In addition, rigid dilators can be costly and may not be readily accessible in all settings [7, 10].

Natural rubber offers greater flexibility, elasticity, and tissue conformity than rigid materials and may theoretically reduce pain, improve tolerability, and lower the risk of mucosal trauma [11]. A soft, flexible dilator that can be safely used at home could improve adherence, caregiver confidence, and clinical outcomes, particularly in resource-limited environments. Natural rubber has long been used in a wide range of medical devices; however, there is still no established standard for an anal dilator that is user-friendly and suitable for self-dilation at home [12].

ParaSelf is a novel natural rubber–based anal dilator designed to better conform to post reconstructive pediatric anal anatomy and facilitate home-based self- or caregiver-administered dilation. However, to our knowledge, natural rubber-based anal dilators have not been systematically evaluated in pediatric patients following anorectal reconstruction.

### Objective

The objective of this study was to evaluate the efficacy and safety of ParaSelf, a natural rubber-based anal dilator, compared with conventional rigid dilators in children after anorectal surgery for ARM or HD.

## Materials and Methods

### Study design

A retrospective pre-post (historical-controlled) cohort study compared outcomes in the ParaSelf implementation period (2022–2025) with those in the preimplementation period using conventional dilators (2017–2022).

### Setting

The study was conducted at Sawanpracharak Hospital, a tertiary care center. Patient data, including clinical characteristics and treatment details, were retrospectively obtained from the electronic medical record (EMR) system.

### Participants

**Figure 1:**
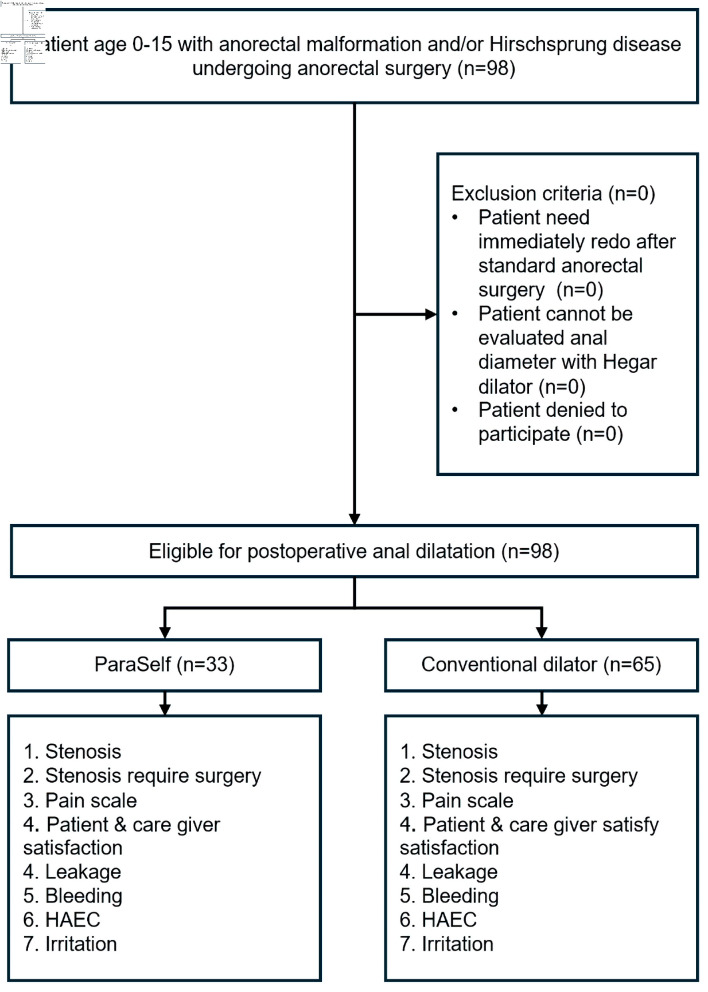
Study flow diagram. HAEC: Hirschsprung-associated enterocolitis.

Patients aged 0–15 with ARM and/or HD who underwent anorectal surgery for correction were included. Patients who required immediate redo surgery after the initial anorectal procedure, those whose anal diameter could not be assessed using a Hegar dilator, and those who declined to participate, were excluded (Fig. 1).

### Intervention

#### Biocompatibility testing (skin irritation tests in rabbits)

To evaluate the cutaneous biocompatibility of the ParaSelf natural rubber anal dilator for pediatric patients, a primary skin irritation test in rabbits was conducted at an International Organization for Standardization (ISO)/International Electrotechnical Commission (IEC) 17025:2017-accredited laboratory, in accordance with ISO 10993-23:2021. Solid, noncolored natural rubber sheets representing the device material (2.5 × 2.5 cm, 20 pieces) were applied as patches to the clipped dorsal skin of three rabbits, with contralateral untreated sites serving as controls. Erythema and edema were scored at 24, 48, and 72 h using the ISO 0–4 grading scale, and Primary Irritation Scores (PIS) and the Primary Irritation Index (PII) were calculated. The mean PII was 0.4, classified as “negligible irritant,” and the test article was concluded not to produce skin irritation in rabbits, supporting the local biocompatibility of the device material [13].

#### Device development

**Figure 2:**
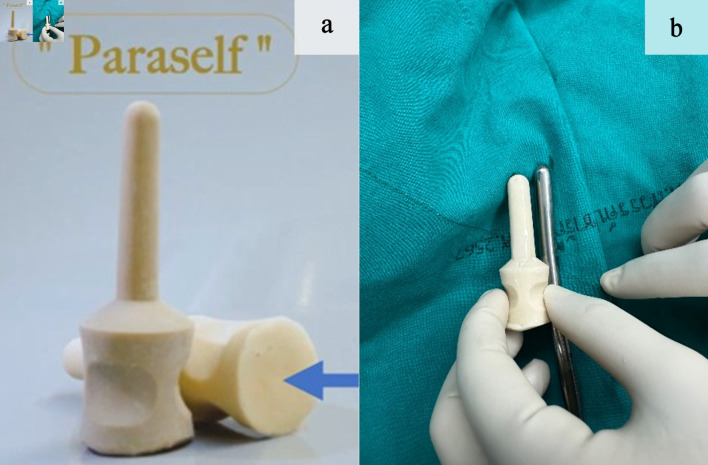
(a) Normal and decubitus positions demonstrating the base (blue arrow). (b) Comparison with Hegar dilators of the same shaft diameter.

ParaSelf is an anal dilatation device made of medical-grade natural rubber with a soft and flexible texture. The device has a cylindrical shaft similar in shape to a Hegar dilator, with a smoothly rounded tip at the insertion end and a flared base that is wider than the shaft. This flared base works as a depth stop to prevent over-insertion and also carries the depth marks and size labels. Below the base, a cylindrical handle with shallow grooves is integrated to improve grip, ending in a broad flat end that supports the hand during use. ParaSelf is produced in several sizes, with shaft diameters ranging from approximately 6 to 20 mm; the height of the shaft increases with larger diameters. Device surface is polished and coated with frictionless substance (Fig. 2a).

The dimensions and proportions of ParaSelf were designed specifically for children and for different postoperative stages. The smallest size has an overall length of about 6 cm, with an insertion segment of 4 cm and a shaft diameter of 2.4 cm, and it is intended for use soon after surgery or in very young children. In clinical use, however, ParaSelf size is selected primarily according to the shaft diameter, and the insertion length and overall length are adjusted proportionally to match each diameter. For consistency with standard reporting, ParaSelf sizes were recorded using the corresponding Hegar shaft diameter as the size reference (Fig. 2b).

The intervention consisted of home anal self-dilation using the ParaSelf natural-rubber dilator. Caregivers received standardized instruction on insertion technique, lubrication, hygiene, and a stepwise schedule for size escalation toward the age-specific target anal diameter. Dilation was performed one to two times daily, with advancement to the next size according to the predefined protocol (with temporary holding/de-escalation when excessive pain, resistance, or bleeding occurred), and anal caliber was assessed at each follow-up visit using Hegar dilators. Estimated dilator costs in our setting were as follows: ParaSelf, 25 Thai Baht (THB) per unit; Hegar dilators, 30,000 THB per set; silicone dilators, 4,000 THB per unit; and resin dilators, 120 THB per unit. These costs are based on local procurement/production estimates and were not derived from a formal economic evaluation.

ParaSelf is a natural-rubber anal dilator manufactured by Friendly Latex Pillow Co., Ltd., Thailand. Although the device was developed by the study team, the device used in this study was manufactured by the above company. At the time of this study, ParaSelf had not yet obtained Thai Food and Drug Administration (FDA) product registration/licensing. The device was used under institutional ethics approval for the present study, and this should not be interpreted as formal regulatory approval for market use.

#### Use of ParaSelf

**Figure 3:**
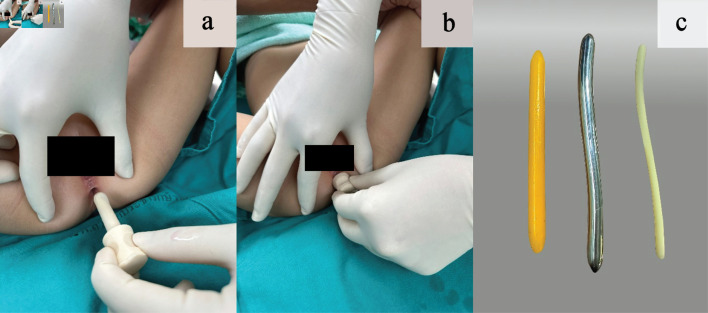
(a) Lubricated rubber-based dilator. (b) The stopper positioned at the neck of the dilator, abutting the perianal skin to prevent over-insertion. (c) Comparison of dilator materials: paraffin-based dilator (yellow), metal-based dilator (silver), and plastic-based dilator (white).

Use of ParaSelf begins with hand washing, cleaning the device, and applying a water-based lubricant. The child is positioned in either the lateral decubitus or supine position (Fig. 3a). The device is then inserted slowly along the natural curve of the anal canal until the depth landmark reaches the skin level (Fig. 3b). The device is kept in anal canal for about 20–30 s to allow the sphincter muscle to relax, and is then withdrawn gently. The recommended frequency is 1–2 times per day for 4–12 weeks [14–16], with gradual stepwise increase in device size according to the schedule. Dilator size escalation followed a stepwise schedule and was surgeon-guided at each follow-up visit, with advancement to the next size based on tolerance and resistance. The insertion depth was standardized according to the ParaSelf specifications; the shaft length (and thus the intended insertion depth) was selected and prescribed by the surgeon. Pain, resistance, or bleeding during home dilation was monitored by caregivers, who were instructed to observe symptoms and signs at each session. The dilation session was temporarily stopped if any of these occurred. If the child refused dilation, the caregiver and patient were re-instructed regarding the risks and benefits of the program, and dilation was resumed when tolerated.

#### Cleaning and storage

After each use, it is rinsed with clean water or mild soap and then air-dried in a well-ventilated area. High heat or strong chemical disinfectants are not recommended, as they may damage the rubber material. Because of the durability of natural rubber, the device can be used repeatedly and remains stable in shape for up to 5 years under normal home use.

#### Standard care

Conventional dilators included paraffin-based dilators (wax candle), metal-based dilators (Hegar dilators), and plastic-based dilators (Fig. 3c) [7]. This composite set could be assigned to any patient, and the assigned dilator type was used throughout the entire dilation program to support adherence and simplify caregiver instruction.

### Endpoint measures

The primary endpoint was the time to reach the target anal diameter (Supplementary Material 1, gr.elmerpub.com) [15, 16]. Dilator size incremental schedule was applied, and anal diameter was measured using Hegar dilator in every visit. Because ARM/HD is associated with a high prevalence of other anomalies, we also adjusted the target anal diameter for age and weight in patients with growth restriction due to any other condition. The initial and final Hegar dilator sizes were measured using the same standard set of instruments. The Hegar dilator served as the standard measuring instrument for anal caliber and anastomotic patency assessment and for routine anorectal examinations in all patients; it was not used as the dilation device in the ParaSelf group. All pediatric surgeons in the anorectal clinic were trained to perform anal caliber measurement using a standardized technique. Time to reach the target anal diameter was measured from the date the postoperative anal dilatation program was initiated (started immediately postoperatively, before hospital discharge) to the date the target anal diameter was first achieved.

Secondary endpoints were: 1) Anal stenosis, defined as failure of the anal diameter to increase with age within 8–10 visits [3]; 2) Anal stenosis requiring surgery, defined as a decrease in anal diameter within 8–10 visits that necessitated operative management (including surgical dilation). For ARM, operative management included serial surgical dilations (under anesthesia, as indicated), redo anoplasty, redo posterior sagittal anorectoplasty, redo anterior sagittal anorectoplasty, V–Y advancement flap, and/or colostomy. For HD, operative management included serial surgical dilations (under anesthesia, as indicated), transanal redo, laparoscopic-assisted redo pull-through, posterior myectomy, cuff excision, and/or colostomy [3]; 3) Pain during self-dilation, assessed using the Face, Legs, Activity, Cry and Consolability (FLACC) scale for children under 3 years of age and the Wong–Baker FACES Pain Rating Scale for children aged 3 years and above [17, 18]. Caregivers and nurses were instructed to monitor and document symptoms and to record pain scores using the appropriate scale; 4) Caregiver satisfaction, assessed using a 5-point Likert scale (5 = strongly agree/very satisfied, 4 = agree, 3 = neutral, 2 = disagree, 1 = strongly disagree/very dissatisfied). The questionnaire was administered by an ARM clinic nurse who was not the surgeon prescribing the dilation program and was independent of the attending surgeon performing anal caliber follow-up; 5) Anastomotic leakage, assessed by physical examination; 6) Bleeding, assessed by physical examination; 7) Hirschsprung-associated enterocolitis (HAEC), assessed by physical examination, which was evaluated in patients with HD only. HAEC was assessed at each follow-up visit (and at interim presentations) and classified as HAEC when the HAEC score was ≥ 10 [19]; 8) Anal irritation: assessed by history taking and physical examination.

### Data sources and measurement

We retrospectively reviewed patients who participated in a postoperative anal dilation program using a conventional dilator (2017–2022) or ParaSelf (2022–2025). Patient data were obtained from medical records and follow-up documentation. Because patients were systematically contacted and closely followed throughout the study period, there were no losses to follow-up or withdrawals. All study variables were fully recorded, with no missing data.

### Study size estimation

Based on pilot data, we expected the cumulative probability of postoperative anal stricture to be 0.40 in patients receiving the conventional dilator and 0.15 in those receiving the ParaSelf device. Using an exponential survival model with a two-sided alpha of 0.05, 80% power, and an allocation ratio of 1:1.5 (ParaSelf vs. conventional), and because the planned follow-up extends beyond 5 years, the sample size was inflated by 20% to account for potential loss to follow-up, yielding final target sample sizes of 57 patients in the conventional group and 38 in the ParaSelf group.

### Statistical analysis

Categorical data were described with frequency and percentage, tested by exact probability test. Normally distributed continuous data were described with mean and standard deviation, tested by independent *t*-test. Non-normally distributed continuous data were described with median and interquartile range, tested by Mann–Whitney U test. In all analyses, statistical uncertainties are expressed in 95% two-sided confidence intervals (CIs). A P value of < 0.05 will indicate statistical significance. Sex was determined based on external genital examination, performed as part of the standard physical evaluation. Continuous variables were analyzed in their original scale without arbitrary categorization. Risk difference regression was used to estimate adjusted risk differences for anal stenosis, anal stenosis requiring surgery, anastomotic leakage, bleeding, HAEC, and anal irritation. Multivariable median regression was used to estimate adjusted median pain scores during self-dilation and caregiver satisfaction. A multivariable Weibull proportional hazards parametric survival model was used to estimate adjusted hazard ratios (HRs) and to generate adjusted failure curves [20]. Unadjusted Kaplan–Meier failure estimates, smoothed hazard estimates, and Schoenfeld residual plots were used to assess potential violations of the proportional hazards assumption; the Schoenfeld residual test indicated that the assumption was acceptable. All endpoints were analyzed using multivariable Weibull survival regression models adjusted for potential confounders, including sex, age, body weight, anatomical subtype [21–23], and baseline anal diameter. The anatomical subtype for ARM was classified according to the Krickenbeck classification, and HD was categorized as low- versus high-type [21, 23]. Stratified analyses by diagnosis (ARM and HD) were performed using multilevel multivariable Weibull parametric survival regression.

### Ethical approval

The study protocol was approved by the Institutional Review Board (IRB) of Sawanpracharak Hospital (Certificate of Approval (COA) No. 14/2568). For the retrospective component, informed consent was waived by the IRB. For the prospective ParaSelf component, all participants (and/or their legal guardians) were informed about the device and the anal dilatation program and provided written informed consent prior to use. This study was conducted as a retrospective intervention study based on routine clinical practice and was not designed or conducted as a clinical trial; therefore, trial registration was not applicable. The study was conducted in compliance with the ethical standards of the responsible institution on human subjects as well as with the Helsinki Declaration.

## Results

A total of 98 patients were included: 33 (33.7%) received ParaSelf and 65 (66.3%) received conventional dilators. Baseline characteristics—including sex, age, body weight, anatomical subtype, and initial anal diameter—were comparable between groups. Although no statistically significant differences were observed, these prognostic variables were prespecified as potential confounders and were adjusted for in the risk difference regression, median regression, and Weibull parametric survival models (Table 1).

**Table 1:** Baseline Clinical Characteristics

Prognostic factors	ParaSelf (n = 33), n (%)	Conventional dilator (n = 65), n (%)	P value
Male	23 (69.7)	44 (67.7)	1.000
Age (month), median (IQR)	6 (1, 48)	23 (1, 45)	0.406
Weight (kg), median (IQR)	8 (4, 12)	8 (3, 12)	0.973
Anatomical subtype			0.755
Short type HD	15 (45.5)	31 (47.7)	
Long type HD	5 (15.2)	13 (20.0)	
Perineal fistula	7 (21.2)	7 (10.8)	
Vestibular fistula	2 (6.1)	5 (7.7)	
Rectourethral fistula bulbar	1 (3.0)	1 (1.5)	
Rectourethral fistula prostatic	1 (3.0)	1 (1.5)	
ARM without fistula	2 (6.1)	7 (10.8)	
Initial anal diameter (mm), median (IQR)	12 (7, 14)	12 (9, 15)	0.128

HD: Hirschsprung disease; ARM: anorectal malformation; IQR; interquartile range.

No missing data were observed. There were no losses to follow-up or withdrawals.

The primary endpoint—time to reach the target anal diameter—was analyzed using a proportional hazards model and reported as an adjusted HR; assessment of Schoenfeld residuals indicated that the proportional hazards assumption was acceptable (Supplementary Material 2, gr.elmerpub.com).

**Figure 4:**
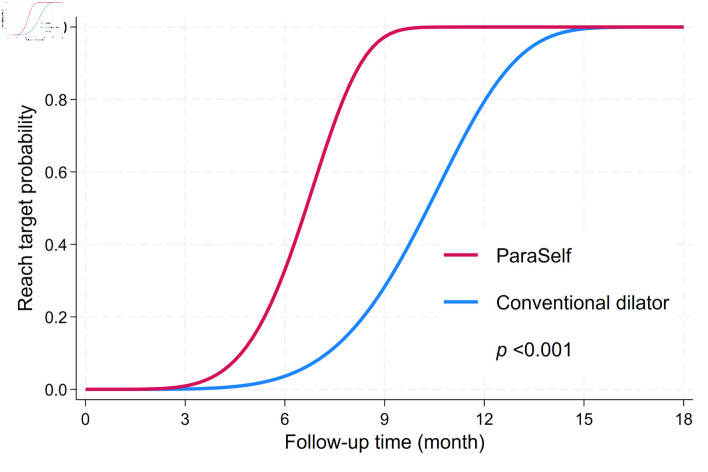
Weibull proportional hazard regression on time to reach target anal diameter.

For the primary endpoint, the ParaSelf group reached the target anal diameter faster than controls (median 5.6 vs. 9.4 months). In the adjusted Weibull proportional hazards model, ParaSelf was associated with a higher hazard of reaching the target diameter HR = 9.9; 95% CI, 5.2–18.7; P < 0.001). Smoothed parametric failure estimates, under a Weibull hazard distribution of the time to reach target anal diameter, illustrate that ParaSelf is significantly faster than conventional dilator, particularly in the first year (Fig. 4).

Patient-reported pain during home dilation was lower in the ParaSelf group, while caregiver satisfaction was higher. Regarding postoperative adverse outcomes, events such as irritation, bleeding, anastomotic leakage, and surgery-requiring stenosis were not observed in the ParaSelf group, and overall complication patterns favored ParaSelf compared with conventional dilators (Table 2). Crude analyses showed that no cases of anal stenosis, surgery-requiring anal stenosis, anastomotic leakage, bleeding, or anal irritation were observed in the ParaSelf group. In the conventional group, 43.1% experienced stenotic events. All patients who developed stenosis required reoperation and/or additional interventions (e.g., redo anoplasty) before they could resume the stepwise dilation schedule (Supplementary Material 3, gr.elmerpub.com).

**Table 2:** Adjusted Risk Differences for Postoperative Endpoints

Endpoint	Clinimetric	Point estimate	95% CI	P value
Anal stenosis	RD	–0.34	–0.48, –0.21	< 0.001
Anal stenosis requiring surgery	RD	–0.08	–0.14, –0.02	0.011
Pain scale during self-dilation	Median difference	–2.58	–3.40, –1.76	< 0.001
Caregiver satisfaction	Median difference	2.00	1.52, 2.48	< 0.001
Anastomotic leakage	RD	–0.06	–0.11, –0.01	0.017
Bleeding	RD	–0.39	–0.53, –0.25	< 0.001
HAEC	RD	–0.16	–0.32, 0.00	0.057
Anal irritation	RD	–0.20	–0.30, –0.11	< 0.001

CI: confidence interval; RD: risk difference; HAEC: Hirschsprung-associated enterocolitis.

All patients ultimately reached the target anal diameter, although some experienced stenotic events during follow-up. At the data cut-off (December 2025), 100% of patients in both groups had achieved the target anal diameter. In adjusted Weibull survival regression, the ParaSelf group reached the age-specific target anal diameter earlier than the conventional group (HR = 9.9; 95% CI, 5.2–18.7), with the greatest separation occurring in the first 3–12 months. No stenotic events were observed in the ParaSelf group (Fig. 4).

Additionally, we performed stratified analyses by diagnosis. The HR for ParaSelf versus conventional dilators was 6.4 (95% CI, 2.1–19.9; P = 0.001) in the ARM subgroup and 10.8 (95% CI, 5.4–21.8; P < 0.001) in the HD subgroup. A multilevel Weibull parametric survival regression including a diagnosis-by-treatment interaction showed no statistically significant heterogeneity of the treatment effect between the ARM and HD subgroups.

## Discussion

ParaSelf can improve the effectiveness of postoperative anal dilatation compared with conventional rigid dilators. Children in the ParaSelf group were more likely to achieve the target anal diameter for age [5], had better continuity of home dilatation, reported substantially less pain [4], and experienced fewer procedure-related complications. Notably, no postoperative anal stenosis was observed in the ParaSelf group, whereas stenosis occurred in 43.1% of children using conventional rigid dilators (e.g., Hegar dilators or rigid resin rods). Given the historical-controlled design, this difference should be interpreted as an association; nevertheless, it suggests that rigid devices may be less favorable for home self-dilation programs in our setting.

Although the target anal diameter was determined according to age-based standards and identical instruction schedules were provided to caregivers in both groups, the practical execution of postoperative anal dilation in daily life is influenced by multiple patient- and caregiver-related factors. In the early postoperative period, particularly in younger children, both physical resistance and psychological distress commonly limit tolerance to anal dilation. Under these circumstances, the use of a softer and more compliant dilator may reduce procedural discomfort and perceived resistance, thereby improving child cooperation and decreasing caregiver anxiety. This combination may facilitate more consistent adherence to the recommended stepwise size progression during the early phase of postoperative care, which, in turn, may contribute to earlier achievement of the target anal diameter, especially within the first year after surgery. Adequate postoperative dilation requires not only a defined target diameter but also sustained cooperation from both the child and caregiver, particularly during the early postoperative phase. Devices that minimize discomfort and resistance may therefore play an essential supportive role in translating standardized protocols into effective real-world practice.

The pathophysiology of anal stenosis in children supports these findings. Infants and young children have a faster wound-healing rate than adults [24]. After anorectal surgery, the anastomotic site or neo-anus quickly enters the proliferation phase, with rapid collagen deposition, extracellular matrix formation, and wound contraction within days. Without regular radial dilatation, this rapid contraction can narrow the anal lumen and progress to fibrosis and stenosis [25]. Previous reports have shown that anastomotic strictures in ARM and HD may begin as early as the first postoperative weeks and are particularly frequent in complex ARM [25]. Therefore, daily anal dilatation during the early postoperative period is essential. Irregular or intermittent dilatation allows repeated cycles of contraction and re-injury, which favor fibrosis and persistent stenosis [26]. There is a narrow margin between causing re-injury and achieving adequate stretching, and this trade-off must be carefully managed. Achieving and maintaining the target anal diameter is thus a key determinant of long-term patency.

The material and design of ParaSelf provide plausible biomechanical advantages over rigid metal or hard-silicone dilators [26]. Conventional Hegar dilators, made of stainless steel or stiff silicone, have a high stiffness and can generate focal pressure and shear forces on the anal mucosa. This may cause micro-tears, mucosal irritation, and local inflammation, which in turn promote collagen overproduction and fibrosis, as reflected by the high stenosis rate in the control group. In contrast, ParaSelf is made of natural rubber with a lower modulus of elasticity, allowing the device to conform to the curvature of the pediatric anal canal and to distribute radial pressure more evenly [27]. This “micro-compliance” results in a form of dynamic dilatation that mimics physiological bowel movements during defecation, reducing shear stress and repetitive trauma to the wound [4]. The absence of stenosis in the ParaSelf group is consistent with this proposed mechanism and with the concept that minimizing local irritation can reduce stricture formation.

Lower pain scores in the ParaSelf group are also clinically important [4]. In this study, children using ParaSelf reported markedly less pain during home dilatation than those using rigid dilators. Reduced pain likely improves cooperation from the child and decreases caregiver anxiety, making it more feasible to perform daily dilatation as prescribed [4]. Better adherence to the dilatation regimen is a known predictor of successful prevention of strictures in ARM and HD, and our findings support the idea that a softer, more tolerable device can translate into improved long-term anatomical outcomes [10].

From an implementation perspective, ParaSelf has a very low per-unit cost (about 25 THB), which may improve accessibility and scalability of home dilation programs compared with commercially available alternatives. However, the cost figures reported here are context-specific estimates rather than results of a formal economic evaluation; therefore, future studies should incorporate standardized cost collection and assess cost-effectiveness alongside hard clinical endpoints (e.g., postoperative stenosis, leakage, and bleeding). In addition, procurement prices and care pathways may vary across settings, and these factors should be considered when interpreting generalizability.

Finally, ParaSelf offers potential benefits at the health system level. By lowering the risk of anal stenosis, the device may reduce the need for repeated dilatation under anesthesia, limit the number of follow-up visits, and decrease hospital resource use. Its low production cost and durability make it suitable for large-scale use, including in resource-limited settings. In addition, ParaSelf supports a family-centered postoperative strategy by enabling safe, acceptable, daily home-based dilatation, which aligns with current trends in pediatric surgical care.

### Limitations, advantages, and future research

Although no missing data were identified in the retrospective dataset, retrospective records may not capture certain potentially important variables. For example, the specific type/model of dilator used may not have been consistently documented, and some devices may have been grouped under the “conventional dilator” category without detailed classification. Because the conventional cohort was drawn from an earlier period, an era effect is possible. Changes over time in surgeon preference, experience and technical expertise, as well as evolving postoperative pathways and nursing care practices, may have influenced outcomes independent of the dilator material. In addition, unmeasured confounders may have influenced both treatment selection and outcomes, leading to residual confounding; a randomized design would best address this limitation. Given the borderline sample size of the present study—particularly for detecting clinically meaningful and statistically reliable differences in secondary outcomes—future adequately powered studies should evaluate hard clinical endpoints such as postoperative stenosis, anastomotic leakage, and bleeding. We could not directly measure protocol adherence because the structured home-dilation implementation and its standardized documentation were introduced only after 2022. Before 2022, conventional dilators were prescribed and used heterogeneously in routine practice, including Hegar dilators, wax candles, resin rods, and plastic dilators. However, Hegar dilator which acts as measurer and dilator applied continuously in measuring the anal diameter.

Finally, the proportional hazards assumption was applied for time-to-event analyses, but this assumption may not hold on theoretical or empirical grounds in this clinical context; future studies should formally test it and consider alternative modeling strategies (e.g., accelerated failure-time models or Laplace regression) when proportionality is violated [28].

### Conclusions

ParaSelf, a soft anal dilator, appears to be a suitable alternative to conventional rigid devices, with lower rates of anal stenosis and postoperative complications, less pain during dilatation, and higher caregiver satisfaction.

## Supplementary Material

Suppl 1 Target anal diameter by age.

Suppl 2 Assessment of the proportional hazards assumption.

Suppl 3 Crude (unadjusted) secondary endpoints.

## Data Availability

The data supporting the findings of this study are available from the corresponding author upon reasonable request.

## Abbreviations

ARMs: anorectal malformations
HD: Hirschsprung disease
PIS: Primary Irritation Scores
PII: Primary Irritation Index
HAEC: Hirschsprung-associated enterocolitis
FLACC scale: Face, Legs, Activity, Cry, Consolability scale
HR: hazard ratio
CI: confidence interval
