# Supplementary material for: Effectiveness of a Natural-Rubber Anal Dilator (ParaSelf) After Pediatric Anorectal Surgery: A Historical-Controlled Intervention Study
Source: Gastroenterology Res. 2026 Apr 27;19(2):100–9. doi: 10.14740/gr2124 (PMC13171265; doi:10.14740/gr2124)
Supplement: Suppl 1 — Target anal diameter by age. [file gr-19-02-100-s001.docx]

**Suppl 1.** Target anal diameter by age

| Age | Diameter  (millimeter) |
| --- | --- |
| <1 month | 6 |
| 1-2 month | 7 |
| 2-3 month | 8 |
| 3-4 month | 9 |
| 4-5 month | 10 |
| 5-6 month | 11 |
| 6-7 month | 12 |
| 7-8 month | 13 |
| 8-12 month | 14 |
| 1-3 year | 15 |
| 3-12 year | 16 |
| >12 year | 17 |
