# Supplementary material for: Effectiveness of a Natural-Rubber Anal Dilator (ParaSelf) After Pediatric Anorectal Surgery: A Historical-Controlled Intervention Study
Source: Gastroenterology Res. 2026 Apr 27;19(2):100–9. doi: 10.14740/gr2124 (PMC13171265; doi:10.14740/gr2124)

**Suppl 2.** Assessment of the proportional hazards assumption: (A) Kaplan–Meier failure estimates, (B) smoothed hazard estimates, and (C) Schoenfeld residual plot.


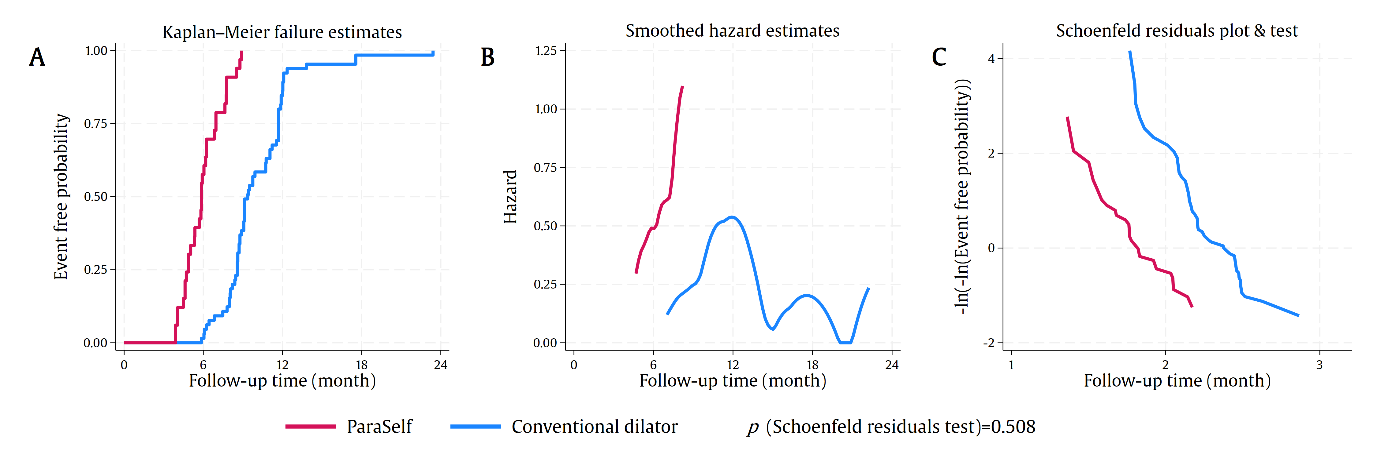

Supplement: Suppl 2 — Assessment of the proportional hazards assumption. [file gr-19-02-100-s002.docx]
