# Supplementary material for: Effectiveness of a Natural-Rubber Anal Dilator (ParaSelf) After Pediatric Anorectal Surgery: A Historical-Controlled Intervention Study
Source: Gastroenterology Res. 2026 Apr 27;19(2):100–9. doi: 10.14740/gr2124 (PMC13171265; doi:10.14740/gr2124)
Supplement: Suppl 3 — Crude (unadjusted) secondary endpoints. [file gr-19-02-100-s003.docx]

**Suppl 3.** Crude (unadjusted) secondary endpoints

| Endpoints | ParaSelf | Conventional dilator | p-value |
| --- | --- | --- | --- |
| Anal stenosis | 0 (0.0) | 28 (43.1) | <0.001 |
| Anal stenosis requiring surgery | 0 (0.0) | 8 (12.3) | 0.035 |
| Pain scale during self-dilation | 1 [1, 1] | 4 [3, 5] | <0.001 |
| Caregiver satisfaction | 5 [5, 5] | 3 [2, 3] | <0.001 |
| Anastomotic leakage | 0 (0.0) | 7 (10.8) | 0.050 |
| Bleeding | 0 (0.0) | 26 (40.0) | <0.001 |
| Hirschsprung-associated enterocolitis | 1 (5.0) | 11 (25.0) | 0.057 |
| Anal irritation | 0 (0.0) | 19 (29.2) | <0.001 |
